# Schleichender demografischer Wandel und neurologische Rehabilitation – Teil 1: Situationsbeschreibung

**DOI:** 10.1007/s00115-022-01415-x

**Published:** 2022-12-19

**Authors:** Stefan Knecht, Harmut Reiners, Mario Siebler, Thomas Platz, Agnes Flöel, Reinhard Busse

**Affiliations:** 1 Institut für Klinische Neurowissenschaften, AG Neurorehabilitation, Heinrich-Heine Universität Düsseldorf, Düsseldorf, Deutschland; 2 Ministerialrat a. D., Ministerium für Gesundheit und Familie (MAGS) Brandenburg, Brandenburg, Deutschland; 3 Fachklinik für Neurologie, Rhein/Ruhr, Essen, Deutschland; 4 Forschung, BDH-Klinik Greifswald, Greifswald, Deutschland; 5 Klinik und Poliklinik für Neurologie, Universitätsmedizin Greifswald, Greifswald, Deutschland; 6 Fakultät Wirtschaft und Management, Technische Universität Berlin, Berlin, Deutschland

**Keywords:** Schlaganfall, Alter, Geriatrie, Erholung, Epidemiologie, Stroke, Old age, Geriatrics, Recovery, Epidemiology

## Abstract

In den nächsten zwei Jahrzehnten werden in Deutschland die Babyboomer aus dem Erwerbsleben ausscheiden. Erwerbsarbeit muss dann von der zahlenschwachen „Pillenknick“-Generation geleistet werden. Mehr ältere Personen in der Gesellschaft bedeuten trotz und teilweise wegen verbesserter medizinischer Möglichkeiten eine höhere Belastung durch Gesundheits- und Pflegeversorgung, die finanziert und personell getragen werden muss. Um mit weniger Erwerbstätigen mehr Bedürftige zu versorgen, muss das Gesundheitssystem umgebaut werden. Weil allerdings die Entwicklungen schleichend verlaufen, ist das Problembewusstsein vielerorts noch gering. Hier fokussieren wir auf den Bereich in unserem Gesundheitssystem, welcher mit am stärksten wächst und zusätzlich den größten Personalbedarf pro Betroffenem hat: die Versorgung schwerkranker und selbsthilfeeingeschränkter Menschen. Das Nebeneinander von Krankenhaus, Rehabilitationsklinik und Pflegeinstitution ist historisch bedingt und unzureichend koordiniert. Es fördert die Tendenz, selbsthilfeeingeschränkte Patient*innen in Pflegeeinrichtungen ohne Chance auf Wiederbefähigung zu entlassen, statt sie zu rehabilitieren. Mit dem weiteren demografischen Wandel droht sich diese Tendenz zu verstärken. Hier versuchen wir in einem ersten von zwei Teilen eine Beschreibung der aktuellen Situation.

## Abstract (en)

In the next two decades the aging baby boomers in Germany will gradually be leaving the work force. They are being followed by the much less numerous, “baby bust” generation who now need to finance and staff healthcare for the growing number of old people in society. In order to care for more needy persons with a smaller working population, the healthcare system must be restructured; however, despite these worrisome prospects, the awareness of the problem is still low in many areas. Here we focus on the area in the healthcare system that is growing particularly rapidly and additionally has the greatest need of personnel per patient: the care of the critically ill and functionally impaired patients. The lack of coordination of hospitals, rehabilitation centers and nursing institution is historical in origin. It promotes the tendency to discharge functionally impaired patients to nursing facilities without giving them a chance for recovery of functional autonomy. As the demographic change progresses, this tendency threatens to increase. In a first of two parts, we attempt to describe the present situation.

## Einleitung

Die Coronavirus Pandemie (SARS-CoV-2) hat noch einmal deutlich gezeigt, dass wir im Gesundheitssystem nicht starr an althergebrachten Vorgehensweisen festhalten müssen, sondern diese zügig an neue Bedürfnisse anpassen können. Zum Beispiel ließ sich – wenn auch nur vorübergehend – regeln, dass Akutpatient*innen in Rehabilitationskliniken behandelt oder Schlaganfallpatient*innen ohne bürokratische Bewilligungsverfahren direkt in die Anschlussrehabilitationen verlegt wurden. Allerdings hätte man rückblickend an vielen Punkten wie z. B. beim Intensivregister schon früher wirksamere Strukturen aufbauen sollen [5].

Jenseits der Pandemie und anderer weltpolitischer Ereignisse besteht eine große Herausforderung fort: der demografische Wandel. Hier sind wir weiter schlecht vorbereitet. Durch seinen schleichenden Verlauf gerät er immer wieder aus dem Fokus. Er trifft besonders die Neuromedizin und ihre derzeitigen Versorgungsstrukturen in Krankenhäusern und Rehabilitationskliniken. Daher hatte die Jahrestagung der Deutschen Gesellschaft für Neurorehabilitation 2020 die Schwerpunktfrage gestellt, wie sich der demografische Wandel auf Medizin und Gesellschaft auswirkt. Dies hat eine anhaltende Diskussion zwischen theoretisch und praktisch Tätigen in Gang gesetzt. Hier berichten wir bisherige Ergebnisse, weil sich unseres Erachtens Gestaltungsmöglichkeiten zeigen, die uns helfen können, die Wucht der demografischen Dynamik teilweise abzufangen, wenn wir die Möglichkeiten früh und beherzt angehen.

Wir beschreiben in einem ersten von zwei Teilen im Folgenden den demografischen Wandel, die Zusammensetzung der Gesundheits- und Pflegeaufwendungen sowie Fehlentwicklungen in der Versorgungskette von Akut- zu Rehabilitationsmedizin.

## Demografischer Wandel

**Figure 1:**
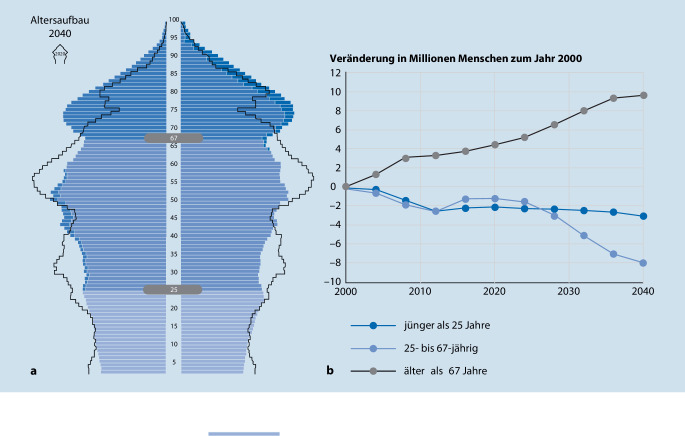

Wir sind in Deutschland mit einer doppelten Alterung konfrontiert. Die Lebenserwartung steigt – allerdings nur noch gering. In den letzten 15 Jahren vor SARS-CoV‑2 nahm sie pro Kalenderjahr nur um 0,1 Jahre zu [14]. Die wichtigere Veränderung ist die Zunahme der Alten an der Bevölkerung. Diese ist zu einem großen Teil dem oft übersehenen dritten Aspekt geschuldet, nämlich dass weniger Kinder als in früheren Generationen geboren wurden. So werden die Jungen nicht nur relativ, sondern auch absolut weniger (Abb. 1). Jetzt, wo es an der Zeit wäre, dass diese (fehlenden) Kinder in die Gruppe der Erwerbstätigen (25- bis 67-Jährige) nachrückten, wird das Problem in Form akzentuierter Personalknappheit und Pflegenotstand manifest [20]. Es hat sein Maximum aber noch keinesfalls erreicht, sondern beginnt gerade erst (Abb. 1). Wir sind also konfrontiert mit einer demografischen Schere aus immer mehr Alten und immer weniger Berufstätigen.

In 40 Jahren dürfte sich die Schere wieder schließen und die Altersgruppen relativ gleichmäßig verteilt sein. Problematisch wird es aber für die in den nächsten 20 Jahren aus dem Erwerbsleben aus- und in eine krankheitsträchtige Phase einsteigende geburtenstarke Babyboomer-Generation. In diesen 20 Jahren wird sich, wenn unsere Lebenserwartung nicht einbricht, die Zahl der Alten (über 67 Jahre) um über 5 Mio. erhöhen (+32 %). Gleichzeitig wird sich die Erwerbsbevölkerung um knapp 7 Mio. Menschen verringern (−14 %). Die Verbliebenen müssen nicht nur mehr Kranke und Pflegebedürftige versorgen, sondern auch das zugrunde liegende Bruttoinlandsprodukt erwirtschaften und die umlagefinanzierte gesetzliche Krankenversicherung tragen. Das Gesundheitssystem ist somit doppelt betroffen: Bereits jetzt sind mehr als 40 % des Gesundheitspersonals älter als 50 Jahre alt [49]. An diesen Zahlen ist leider nicht zu rütteln, weil alle alten Akteure schon leben und fehlende junge Akteure nicht mehr nachträglich geboren werden können. Migration könnte diese Entwicklung nur kompensieren, wenn sie jedes Jahr netto bei ungefähr einer Million qualifizierten Zuwanderern unter 47 Jahren läge. Das Statistische Bundesamt rechnet in einem moderaten Modell jedoch nur mit einem Migrationssaldo von 220.000 pro Jahr. In einem hoch angesetzten Modell unterstellt es 310.000 Nettozuwanderer. Aktuelle weltpolitische Ereignisse könnten diese Zahl jedoch für kurze Zeit ansteigen lassen.

In der Industrie ließe sich über einen Zeitraum von 20 Jahren mit 14 % weniger Personal 32 % mehr Leistungen erbringen und zwar über Produktivitätssteigerung, v. a. durch Automatisierung. Das Problem der Gesundheits- und Pflegeversorgung ist jedoch, dass sie zu über zwei Drittel Mensch-zu-Mensch-Leistung ist. Ähnlich wie in anderen rationalisierungsresistenten Dienstleistungsberufen wie Frisörhandwerk oder Lehre gibt es in der Medizin weniger Automatisierungspotenzial als im produzierenden Gewerbe [1].

## Rolle von Alter für Gesundheits- und Pflegeaufwendungen

Deutschland gibt bei gut 80 Mio. Einwohnern derzeit bereits jeden Tag über 1,2 Mrd. € für Gesundheit aus (im Jahr 2020). Träger dieser Ausgaben ist größtenteils die Gesetzliche Krankenversicherung, derzeit bestehend aus gut 100 gesetzlichen Krankenkassen und 40 privaten Krankenversicherern. Ungefähr 25 % der Gelder gehen an Krankenhäuser und knapp 15 % an Arztpraxen, ebenfalls 15 % in die Pflege. Weitere 20 % werden für Medikamente und Materialien aufgewandt, der Rest für Labor, Verwaltung, Transport und anderes. Finanziert werden mit diesen Ausgaben pro Jahr knapp 20 Mio. stationäre Krankenhausbehandlungen (zumindest vor COVID-19) von im Mittel 7 Tagen Dauer in etwas unter 2000 Krankenhäusern und über 1 Mrd. Kontakte in über 100.000 Arztpraxen.

Mit zusätzlicher Finanzierung durch Rentenversicherungen werden in Deutschland knapp 2 Mio. stationäre Rehabilitationsbehandlungen in gut 1000 Rehabilitationskliniken durchgeführt ([47] und Datenbankabfrage ebenda per www.gbe-bund.de). Mehr als ein Zehntel dieser Behandlungen sind neurologische Rehabilitationen [15]. Während anderen Fächern eine Aufwandsdifferenzierung fehlt, existiert in der Neurologie eine Einteilung nach Schwere der funktionellen Beeinträchtigungen von Patient*innen, das Phasenmodell der Neurorehabilitation (s. Infobox 1). Dies ermöglicht der Neurorehabilitation – und nur ihr – auch die Rehabilitation schwerer Betroffener (teilweise werden in interdisziplinären Frührehabilitationsabteilungen schwerer Kranke nach neurofrührehabilitativem Vorbild versorgt). So werden in Deutschland pro Jahr zusätzlich zu konventioneller Rehabilitation ungefähr 40.000 Neurofrührehabilitationen bei Schwerstbetroffenen durchgeführt, die noch eine Versorgung auf Intensivmedizinniveau brauchen. Dazu kommen noch einmal 10.000 rehabilitationsneurologische Beatmungsentwöhnungen, kurz Neuroweaning [36]. Die überwiegend in integrierten Neurorehazentren angesiedelten Einheiten für neurologische Frührehabilitation und Neuroweaning fallen meistens in den Krankenhausbereich. Die Neurologie verklammert damit wie kein anderes Fach Akutmedizin und Rehabilitation.

Neben der Krankenversorgung gibt es in Deutschland die Pflegeversorgung mit eigener (Teilkasko‑)Versicherung. Diese deckt Pflegekosten anteilig ab. Der Rest der Kosten verbleibt bei Betroffenen, ihren Familien oder der Sozialhilfe. In Deutschland erhalten 3,4 Mio. Menschen Pflegeleistungen mit einem Marktvolumen von ca. 35 Mrd. € in der stationären und 20 Mrd. € in der ambulanten Pflege [45]. Diese Kosten sind ungefähr so hoch wie die Ausgaben für die Arztpraxen. Pflegebedürftigkeit und deren Kosten sind jedoch das Ergebnis von Gesundheitsversorgung und könnten durch eine verbesserte Behandlung und anschließende Rehabilitation reduziert werden.

Medizin ist eine aufwendige Dienstleistung. Entsprechend entfallen gut zwei Drittel der Kosten in ambulanter und stationärer Versorgung auf Personal. Insgesamt sind in Deutschland im Gesundheitssystem über 600.000 Pflegekräfte und knapp 400.000 Ärzt*innen tätig – neben über 4,5 Mio. anderen Beschäftigten u. a. in medizinassoziierten Industrien [6, 7, 49].

Die Gesundheitsausgaben sind in den letzten 10 Jahren (von 2010 bis 2020) insgesamt um 50 % angestiegen, davon Ausgaben für Pflege um 60 %. Da auch das Bruttoinlandsprodukt gewachsen ist, stieg der Anteil der Gesundheitsausgaben daran jedoch „nur“ von 11,3 % auf 13,1 % [48]. Damit wird jeder 8. in Deutschland umgesetzte Euro im Gesundheitssystem ausgegeben bzw. erwirtschaftet.

**Figure 2:**
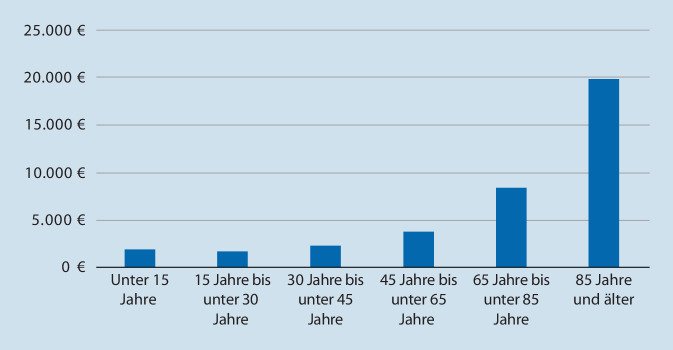

Die Gesundheitsausgaben verteilen sich unterschiedlich auf die Bevölkerung. Das Gros der Ausgaben wird für die über 65-Jährigen getätigt (Abb. 2).

Die Zusammenhänge von Bevölkerungsalterung und Morbiditätslast, und damit die Gesamtkosten durch Krankheit, werden durch unterschiedliche, schwer quantifizierbare Mechanismen beeinflusst [30, 34]. Nach der Expansionsthese der Morbidität ist der Anstieg der Lebenserwartung mit Ausweitung von Zeiten chronischer Krankheit und Pflegebedürftigkeit sowie dadurch vermehrten Gesundheitskosten verbunden. Die Kompressionsthese hingegen unterstellt, dass durch den medizinischen Fortschritt Krankheiten und Pflegebedürftigkeit in immer späteren Lebensjahren auftreten und sich durch eine biologisch limitierte Lebenserwartung sogar verringern. Dies würde eine Verkürzung oder Kompression von Zeiten chronischer Krankheit und Pflegebedürftigkeit bedeuten. Zwischen Expansions- und Kompressionsszenarien steht die These eines dynamischen Gleichgewichts, die einen Anstieg der gesunden wie auch der gesundheitlich beeinträchtigten Lebenszeit erwartet. Nimmt man die ansteigende Zahl der von Niedergelassenen gemeldeten chronischen Erkrankungen in Deutschland, sprechen die Daten für eine Expansion der Morbiditätslast [50]. Nimmt man aber die Patientenselbsteinschätzung der eigenen Gesundheit, sehen wir eine Kompression von Krankheitslast [43].

Zur Bevölkerungsalterung hinzu kommt die Rolle des medizinischen Fortschrittes. Neue Verfahren verursachen zusätzliche Kosten [23], senken aber meist die Krankheitslast. Thrombektomien in der Schlaganfallbehandlung vermeiden Tod und Pflegebedürftigkeit [26]. Aortenklappenersatz erhält Mobilität und vermeidet Pflegekosten. Viele dieser Innovationen kommen besonders Älteren zugute. Obwohl das durchschnittliche Alter der Bevölkerung zwischen 2005 und 2020 nur um 2 Jahre gestiegen ist, waren es bei stationär Behandelten 5 Jahre [19]. Dies zeigt, dass das Gesundheitssystem stärker als früher im Alter genutzt wird.

## Altersassoziierter Bedarf an Intensivmedizin und neurologischer Frührehabilitation

Medizinische Innovationen verbessern zwar das Überleben alter, multimorbider oder kritisch kranker Patient*innen, sie erhöhen dadurch aber auch den Bedarf an aufwändiger Medizin für diese Menschen. So hat sich die Zahl der intensivmedizinischen Behandlungen mit Beatmung in Deutschland bereits vor der SARS-CoV-2-Pandemie über die vorangehenden 15 Jahre auf eine knappe halbe Million verdoppelt [44]. Daten aus Frankreich zeigen, dass sich dort trotz insgesamt abnehmender Krankenhausaufnahmen die Zahl der Intensivbehandlungen ebenfalls annähernd verdoppelt hat und zwar überproportional durch alte Patient*innen. Die Mortalität auf den Intensivstationen hat sich dadurch nicht verschlechtert [32].

Viele – je nach Studie zwischen 25 und 100 % – und vor allem ältere Intensivpatient*innen entwickeln unabhängig von ihrer Grunderkrankung neurologische Funktionseinschränkungen in Form einer Intensivstationsschwäche (auch „ICU-acquired weakness“, Critical-illness-Polyneuropathie/-myopathie o. a. genannt; [29, 42]), häufig verbunden mit kognitiven Leistungsminderungen und emotionalen Belastungen (Ängste und Depressivität), zusammen Post-intensive-care-Syndrom (PICS) genannt, mit erheblichem Gefährdungspotenzial für Selbstversorgungskompetenz [12, 35]. Die Intensivstationsschwäche resultiert aus einer Multiorganschädigung durch ein Zusammenspiel unterschiedlicher Schadfaktoren einschließlich Inaktivität selbst. Nicht nur periphere Nerven sind gefährdet, sondern auch die großen rumpfnahen und normalerweise daueraktiven Muskeln. Das schließt die Atem- und Schluckmuskulatur ein. Systematische Studien zeigen, dass das Zwerchfell bei 80 % der Patient*innen mit Intensivstationsschwäche betroffen ist [24] und eine klinisch relevante Störung der Schluckmuskeln je nach Schwere der Intensivstationsschwäche bei bis zu 100 % der Patient*innen zu finden ist [53].

**Figure 3:**
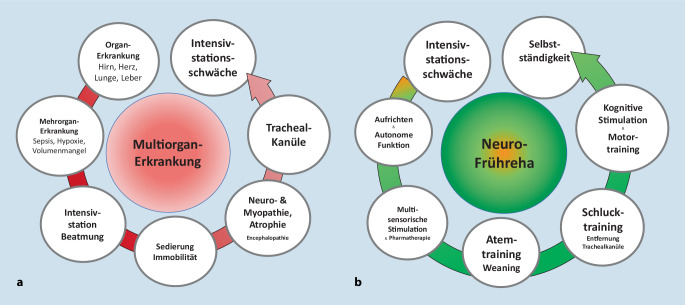

So hat sich wie die Intensivbehandlungen auch die Zahl der neurologischen Frührehabilitationsbehandlungen erhöht. Die Neurofrührehabilitation ist die wesentliche Nachversorgungsform für Patient*innen nach prolongierter Intensivbehandlung. Auf jeden 10. beatmeten Intensivpatient*innen (ca. 20 % der Intensivpatient*innen werden beatmet) kommt eine neurologische Frührehabilitationsbehandlung. Ähnlich wie bei der Rehabilitation anderer Lähmungen werden bei diesen Patient*innen Atem- und Schluckmuskulatur systematisch trainiert und Maschinenunterstützung sukzessive und kontrolliert reduziert (Abb. 3). Eine frühe Neurorehabilitation von Patient*innen verbessert nicht nur die Mobilität bei Entlassung, sondern verlängert auch die Lebenserwartung [51]. Ohne eine neurologische Frührehabilitation und nachfolgende reguläre Neurorehabilitation haben Betroffene wenig Chancen auf Unabhängigkeit von intensivmedizinischen Apparaten.

## Fehlentwicklungen: Pflege vor Rehabilitation

Alte Patient*innen profitieren zwar erheblich von den verbesserten Möglichkeiten der Spitzenmedizin. Sie entwickeln aber besonders leicht immobilitätsbedingte Funktionsbeeinträchtigungen, weil sie weniger Kompensationsreserven haben [42]. So sind Ältere zu Beginn einer Anschlussrehabilitation im Mittel stärker funktionsbeeinträchtigt als Jüngere. Von einer intensiven Neurorehabilitation profitieren sie hinsichtlich des absoluten Funktionsgewinns aber genauso wie Jüngere [28, 31].

**Figure 4:**
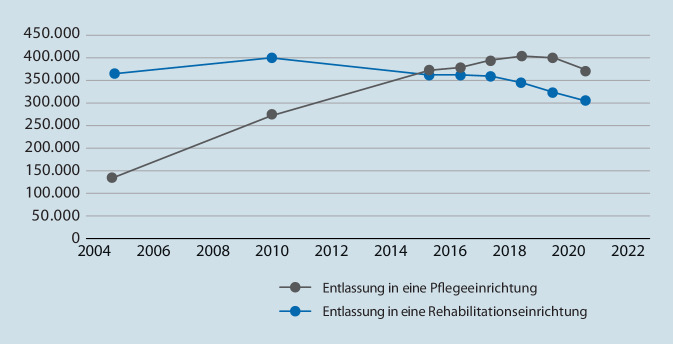

Da auch unsere Sozialgesetzgebung Rehabilitation vor Pflege fordert (§ 31 SGB XI), sollte man angesichts älter und kränker werdender Patient*innen nicht nur einen stärkeren Anstieg der neurologischen Frührehabilitation sehen, sondern einen Anstieg aller Anschlussrehabilitation. Dies ist aber nicht der Fall. Stattdessen ist die Zahl der Anschlussrehabilitation in den letzten 15 Jahren sogar gefallen und die Zahl der Entlassungen aus dem Krankenhaus direkt in Pflegeeinrichtungen hat sich mehr als verdoppelt (Abb. 4). Da wundert es nicht, dass parallel die Zahl der Pflegeheime (mit im Schnitt stabil knapp 60 Gepflegten) seit 2011 von 12.000 auf über 15.000 angestiegen ist [7]. Noch gravierender ist die Entwicklung bei den Entlassungen von Intensivstationen. Hier hat sich innerhalb von 10 Jahren nicht nur der Anteil von Verlegungen in Pflegeheime verdoppelt. Zusätzlich hat sich der Anteil von Weiterverlegungen in Rehabilitationseinrichtungen seit 2010 sogar um ein Viertel vermindert [18].

Pflegeheimunterbringung bedeutet aber Verzicht auf Wiederbefähigung zu einem eigenständigen Leben. Die Tageskosten für eine Pflegeheimversorgung sind zwar geringer als die einer Neurorehabilitation. Neurorehabilitation reduziert aber Pflegebedürftigkeit [31] und spart daher sogar bei über 80-Jährigen Kosten gegenüber einer Pflegeheimeinweisung – außer bei Menschen, die innerhalb von 6 Monaten versterben und daher weniger Pflegekosten verursachen [28]. Die Direktverlegungen von Patient*innen in Pflegeheime verschenken nicht nur Chancen für die Betroffenen und verursachen Kosten, sondern binden auch zusätzliches, an anderer Stelle benötigtes Pflegepersonal. Bereits jetzt geht der Pflegereport (mit dem Untertitel: „Mehr Personal in der Langzeitpflege – aber woher?“) davon aus, dass in den nächsten 20 Jahren der Bedarf an Pflegekräften von 600.000 auf über 800.000 ansteigen wird [6].

Noch eklatanter sind die Versorgungsverzerrungen bei Schwerkranken, die statt in eine spezialisierte Rehabilitationseinrichtung direkt in eine Pflegeeinrichtung mit aufwendiger Krankenpflege (Behandlungspflege nach § 37 SGB V) verlegt werden. Im Jahr 2005 gab es laut Daten der Krankenkassen ca. 1000 so intensivmedizinisch Pflegeversorgte. Diese Zahl hatte sich bereits bis 2017 auf 15.000 bis 30.000 erhöht. Mit jährlichen Behandlungskosten zwischen 250.000 und 360.000 € pro Person entspricht diese Form der Pflege finanziell einer dauerhaften Intensivstationsbehandlung [2, 25]. Der intensivmedizinische Pflegebereich ist nach Betten und Kosten damit annähernd so groß wie der eigentliche Intensivmedizinbereich. Neben den reinen Kosten fällt auch hier die erhebliche Personalbindung ins Gewicht. Benötigt werden nicht nur hochqualifizierte Pflegefachkräfte, sondern eine vollständige 24-stündige Abdeckung. Auch diese Kräfte fehlen im kurativen und rehabilitationsmedizinischen Bereich. Die Deutsche Interdisziplinäre Gesellschaft für Außerklinische Beatmung kommt gemeinsam mit weiteren Fachgesellschaften zu dem Schluss, dass intensivmedizinische Pflegebedürftigkeit in vielen Fällen durch Rehabilitation vermieden werden könnte [16].

Mit dem Intensivpflege- und Rehabilitationsstärkungsgesetz ist den Intensivmedizinern jetzt aufgegeben, bei beatmeten oder tracheotomierten Patient*innen vor Verlegung das Rehabilitationspotenzial zu prüfen [8]. Ob diese Anweisung die Dynamik der verwahrenden intensivmedizinischen Pflege wesentlich ändern wird, bleibt abzuwarten, weil es in vielen Regionen an entsprechenden spezialisierten Rehabilitationseinrichtungen mangelt [9]. In NRW z. B. fehlen über 800 Neurofrührehabetten [21, 22].

## Akteure

Das deutsche Gesundheitsmodell Bismarckscher Prägung ist nicht um Patient*innen und deren Versorgungskette herum organsiert [10]. Es versucht vielmehr, unterschiedliche, aus historischen Kontexten entstandene und eigene Interessen verfolgende Gesundheitsakteure wie Krankenkassen, Krankenhäuser und Rehabilitationskliniken zu koordinieren. Dazu kann, meist auf Bundesebene und vermittelt durch den Gemeinsamen Bundesausschuss, die Politik den Regulationsrahmen anpassen [40]. Grundlegende Organisationsstrukturen sind aber schwer zu ändern.

Eine solche grundlegende Struktur ist die Trennung von Krankenhaus und medizinischer Rehabilitation. Für die Versorgung alter und multimorbider Patient*innen wäre eine besonders effiziente Versorgungskette nötig. Dafür sollten zumindest Krankenhaus- und Rehabilitationsplanung abgestimmt sein. Dies ist aber nicht der Fall. Krankenhausplanung ist Aufgabe der Länder. Bereitstellung stationärer Rehabilitation ist aber nach § 40 SGB V zunächst Aufgabe der Krankenkassen. Diese Bereiche greifen nicht ineinander.

### Länder

Der Krankenhausbereich wird direkt von den Ländern verantwortet, bedarfsanalysiert und beplant. Die Planungen erlauben auch neue Wege wie derzeit in Nordrhein-Westfalen die Festlegung medizinischer Leistungsbereiche und -gruppen in Kombination mit Krankenhausstrukturvorgaben. Diese Planung stößt aber an Grenzen, wenn es um zunehmend ältere und kränkere Menschen geht. Weil ältere Menschen weniger Kompensationsreserven haben, reicht für viele unter ihnen eine Akutversorgung nicht aus. Sie benötigen häufiger funktionsunterstützende und (früh-)rehabilitative Therapien. Eine auf eine alternde Bevölkerung ausgerichtete Krankenhausplanung bräuchte daher eine abgestimmte Frührehabilitations- und Rehabilitationsplanung. Letztere obliegt aber nicht primär den Ländern, sondern den Krankenkassen. Nur wenige Länder wie Bayern positionieren sich normativ, indem sie in der Neurologie neben der in die Krankenhausplanung fallenden Frührehabilitation alle Phasen der Rehabilitation unter einem Dach als planungsrechtliches Kernelement definieren [3].

### Krankenkassen

Eine Rehabilitationsplanung der Krankenkassen, vergleichbar der Krankenhausplanung der Länder, gibt es nicht. Die Krankenkassen verlassen sich bei der Bereitstellung stationärer Rehabilitation auf das Spiel des Anbietermarktes, indem sie mit Bewerbern selektive Versorgungsverträge schließen oder fallbezogene Einzelabsprachen treffen. Bedarfe werden aber nicht definiert und Bedarfsdeckungen wird nicht gesteuert. Dies führt gerade in der Anschlussrehabilitation zu Versorgungsdefiziten. Diese sind wesentlicher Grund, warum mittlerweile mehr Krankenhauspatient*innen direkt in Pflegeheime verlegt werden als in Rehabilitationseinrichtungen, obwohl Reha vor Pflege gilt. Auf der Basis der Krankenhausentlassdaten in Abb. 4 ergibt sich für die Anschlussrehabilitation ein doppelt so hoher Kapazitätsbedarf, wie bisher gedeckt ist [19]. Unter Berücksichtigung eines Anteils Moribunder und mittlerer Verweildauern in der Rehabilitation wären das ungefähr 30.000 Anschlussrehabilitationsbetten, die fehlen. Dieses Defizit wird in der Praxis den Beteiligten aber nicht direkt ersichtlich, weil vor der Anschlussrehabilitation ohnehin der Flaschenhals des Bewilligungsverfahrens steht. Dieser aus der Kurmedizin und der beruflichen Rehabilitation stammende Prozess zur Beantragung einer Rehabilitation bei den jeweiligen Kostenträgern ist häufig mit langen Wartezeiten verbunden. Unter Zeit- und Bettendruck stehende Krankenhäuser werden dadurch geradezu gedrängt, den Weg zur Direktverlegung in Pflegeheime zu wählen – zulasten der Chancen von Patient*innen auf Wiedererlangung von Selbstständigkeit, aber auch zulasten der Pflegekassen. Obwohl Aufwendungen für Krankenhausbehandlung und medizinische Rehabilitation einerseits und Pflegeversorgung andererseits bei den Krankenkassen angesiedelt sind, werden sie dort separat verwaltet. Daher fordern Gesundheitswissenschaftler schon lange eine effektive Zusammenlegung von Kranken- und Pflegeversicherung [17].

### Krankenhäuser

Für Krankenhausmitarbeiter ist die einfachste und schnellste Entlassart für noch nicht wieder selbsthilfefähige Patient*innen, wenn noch akuter Krankenhausbedarf geltend gemacht werden kann, die Verlegung in geriatrische Kliniken, in denen sie oft keine für Funktionserholung nach Hirnschädigung ausreichende Rehabilitation bekommen, oder, wenn kein Krankenhausbedarf mehr geltend gemacht werden kann, in Pflegeheime ohne Rehabilitation. Für beide Konstellationen gibt es keine aufwendigen, mit Rückfragen oder Verzögerungen behafteten Prüfverfahren. Ein Teil der zunächst in geriatrischen Kliniken weiterbehandelten Patient*innen kommt danach in neurologische Rehabilitationskliniken, ein anderer wird in Pflegeheime verlegt. Direktverlegungen in Pflegeheime werden jetzt noch einmal beschleunigt durch die erleichterte Auffindbarkeit verfügbarer Pflegeheimplätze über elektronische Matching-Plattformen. Bemühungen, Patient*innen über die Barrieren hinweg in Anschlussrehabilitationen zu verlegen, führen zu verlängerten Liegezeiten und verschlechtern dadurch das Betriebsergebnis der Krankenhäuser.

Unser Gesundheitssystem fördert daher eine Medizin, die ältere Kranke hervorragend akutmedizinisch versorgt, sie aber dann auf der zweiten Hälfte der Strecke auf dem Weg zurück zur Selbständigkeit im Stich lässt.

### Rehabilitationskliniken

Stationäre Rehabilitation ist organisatorisch noch stark vom Kurwesen geprägt. Qualitätsprüfungen erfolgen wie bei Kundenzufriedenheitserfassungen in der Hotellerie über Patientenbefragungen statt über die naheliegende Messung der risikoadjustierten Verbesserung von Selbsthilfe- und Teilhabefähigkeit. Rehabilitationsanbieter versuchen, stationäre Rehabilitationsleistungen zu einem für sie günstigen Kosten-Preis-Verhältnis an ihre Kunden, die Kassen, zu verkaufen. Das sind vor allem Rehabilitationsleistungen für medizinisch stabile und wenig unterstützungsbedürftige Patient*innen. Entgelte werden einrichtungsspezifisch verhandelt und immer in Konkurrenz zur Nachbarklinik. Therapiemengen, insbesondere fachlich erforderlicher spezifischer Therapie spielen eine nachgeordnete Rolle [37, 38].

**Figure 5:**
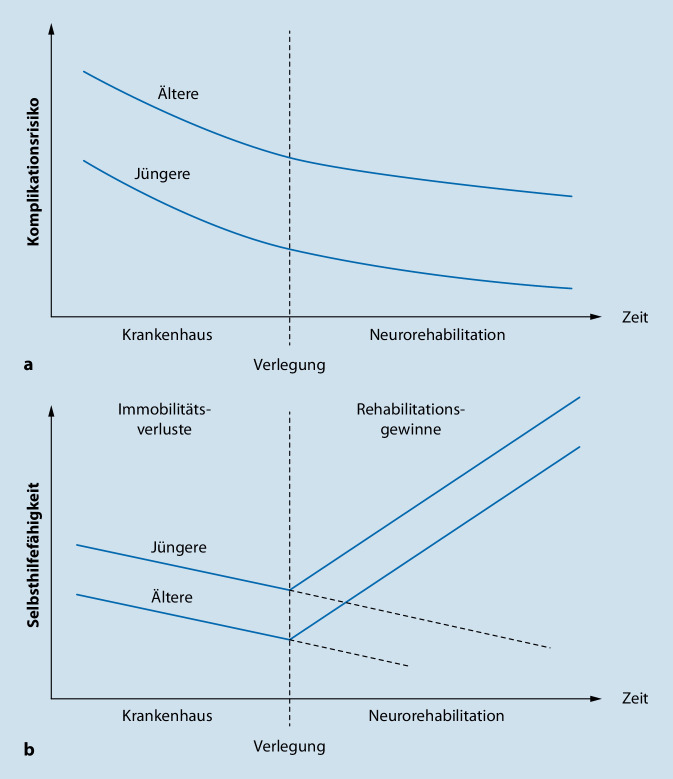

Die meisten Rehabilitationsbehandlungen in Deutschland sind von den Rentenversicherungen beauftragt. Ungefähr ein Viertel der stationären Rehabilitationen jedoch sind Anschlussrehabilitationen nach Krankenhausaufenthalt, zumeist im Auftrag der Krankenkassen. Bei ungefähr der Hälfte davon handelt es sich um Neurorehabilitationen. Trotz ihres Phasenmodells kommt aber auch die Neurorehabilitation mit den älter und kränker werdenden Patient*innen an ihre Grenzen, weil das Modell nur Pflegebedürftigkeit, aber nicht Morbidität berücksichtigt. Ältere und multimorbide Patient*innen brauchen jedoch umfangreiche zusätzliche personelle und apparative medizinische Vorhaltungen [27]. Eine längere vorgeschaltete Krankenhausbehandlung löst das Problem leider nicht, weil das Komplikationsrisiko bei Älteren nur sehr langsam abfällt und gleichzeitig Patient*innen in Krankenhäusern zunehmende immobilitätsbedingte Funktionsverluste erleiden (Abb. 5).

### Vertragsärztlicher ambulanter Bereich

Auch nach einer stationären Rehabilitation verbleiben oftmals Selbsthilfe- und Teilhabeeinschränkungen, zu deren Rückbildung Patient*innen weitere gezielte und v. a. koordinierte rehabilitative Behandlung unter Einschluss mehrerer therapeutischer Disziplinen bräuchten [4, 52]. Ambulant werden jedoch meist isolierte Therapieserien verschrieben. Häufig müssen Verschreibungen auch erst von den Betroffenen selbst angestoßen werden. Dieses Vorgehen greift regelhaft zu kurz, weil das notwendige integrierte Konzept fehlt [31]. Dies liegt nicht nur an mangelnder einschlägiger Rehabilitationskompetenz, sondern auch am Fehlen passender (Vergütungs‑)Strukturen.

Um eine teambasierte ambulante rehabilitative Behandlung etablieren zu können, bräuchte es Ausbildung, den Einbezug neurologischer (Früh‑)Rehabilitationszentren und die Etablierung zugehöriger Vergütungsstrukturen. Ein Beispiel ist das bayrische Gesundheitsprojekt „OptiNIV“ (Akronym für die „Optimierung der nachklinischen Intensivversorgung bei neurologischen Patienten“; www.optiniv.de). Patienten mit Bedarf an maschineller Beatmung und/oder Trachealkanülenversorgung und damit für die außerklinische Intensivpflege werden nach ihrer Entlassung aus der Neurofrührehabilitation regelmäßig durch Teams aus den neurologischen Frührehabilitationseinrichtungen nachuntersucht. Die Teams stellen individuelle Managed-care-Pläne für die ambulant wohnortnah zuständigen medizinischen Fachkräfte zur Verfügung. Sie führen kurze stationäre Untersuchungen und ggf. eine stationäre Wiederholungsrehabilitation in den jeweiligen neurologischen Frührehabilitationseinrichtungen durch, wenn während der Nachsorge ein Entwöhnungspotenzial von mechanischer Beatmung und/oder Dekanülierung beobachtet wird. Durch diese transektorielle Betreuung soll durch ein „continuum of care“ das Langzeitergebnis (Entwöhnung von Beatmung und/oder Trachealkanüle) und damit die Abhängigkeit von außerklinischer Intensivpflege reduziert werden.

Hemmend für effektive ambulante Rehabilitation ist auch, dass die Vergütung sich an „traditionellen“, nichtleitliniengerechten Positionen ausrichtet, während gleichzeitig Behandlungsformen mit belastbaren Wirksamkeitsnachweisen gar nicht oder schlecht abgebildet werden, z. B. durch fehlende Berücksichtigung von Investitionsaufwendungen für spezifische Geräte wie etwa Rehabilitationsrobotik. Besonders schwierig ist poststationäre Versorgung im ländlichen Raum.

#### Infobox 1

Krankenhäuser:

- Verantwortung bei Ländern
- Aufnahmeentscheidung bei Ärzten
- Entgelte v. a. über mit durchschnittlichen Kosten kalkulierte Pauschalen (DRGs)

Rehabilitationskliniken:

- Verantwortung bei Kostenträgern (v. a. Renten- und Krankenversicherung)
- Aufnahmeentscheidung bei Kostenträgern und Kliniken, mittlerweile teilweise algorithmisch
- Entgelte verhandelt zwischen Einzelklinik und Kostenträger, der zusätzlich Patientenzuweisung steuert

Neurorehabilitation:

- Findet statt in Krankenhäusern, Rehabilitationskliniken und integrierten Neurorehazentren (Kombination aus Krankenhaus und Rehaklinik)
- Phasenmodell mit Ungefährbeschreibungen
  - A: Akutbehandlung (Krankenhaus)
  - B: überwachungs- oder aufwendig pflegebedürftig (meist Krankenhaus, teilweise Rehaklinik)
  - C: bettlägrig oder rollstuhlbedürftig (Rehaklinik)
  - D: selbsthilfefähig (Rehaklinik)
- Neurofrührehalitation (neurologisch-neurochirurgische Frührehabilitation)
  - Krankenhausbehandlung entsprechend OPS 8552 oder Beatmungs-DRG (verstehbar als Teilmenge der Neurorehaphase B)
- Anschlussrehabilitation
  - Rehabilitation innerhalb von 14 Tagen nach Krankenhausbehandlung
  - Anschlussheilbehandlung (AHB) als Sonderform der Anschlussrehabilitation für schon wieder weitestgehend selbsthilfefähige Patient*innen

Pflege – Teilleistung durch Pflegeversicherung in 5 Pflegegraden, Rest durch Betroffene:

- Ambulant
  - Angehörige oder Pflegedienste
  - „24-Stunden-Pflege“: Betreuung durch meist nichtexaminierte Person, die in Hausgemeinschaft lebt und auch für Bereitschaftszeit bezahlt werden muss
  - Außerklinische Intensivpflege: Versorgung mit ständiger Anwesenheit einer geeigneten Pflegefachkraft für Patient*innen mit Tracheostoma oder Beatmung (§ 37c SGB V) meist in Wohngemeinschaften
- Stationär
  - Einrichtungseinheitliche Eigenanteile für Betroffene mit Pflegegraden 2 bis 5
